# The Effect of Ethanol on Abnormal Grain Growth in Copper Foils

**DOI:** 10.3390/nano11113069

**Published:** 2021-11-15

**Authors:** Zhancheng Li, Yongna Zhang, Yinwu Duan, Deping Huang, Haofei Shi

**Affiliations:** 1 Chongqing Institute of Green and Intelligent Technology, Chinese Academy of Sciences, Chongqing 400714, China; lizc@cigit.ac.cn (Z.L.); zhangyn@cigit.ac.cn (Y.Z.); 2 Chongqing Engineering Research Center of Graphene Film Manufacturing, Chongqing 401329, China; duanyw@moxigroup.com

**Keywords:** abnormal grain grown, decimeter-size grains, ethanol annealing atmosphere, recrystallization of copper foils, contact-free annealing

## Abstract

Single-crystal Cu not only has high electrical and thermal conductivity, but can also be used as a promising platform for the epitaxial growth of two-dimensional materials. Preparing large-area single-crystal Cu foils from polycrystalline foils has emerged as the most promising technique in terms of its simplicity and effectiveness. However, the studies on transforming polycrystalline foil into large-area single-crystal foil mainly focus on the influence of annealing temperature and strain energy on the recrystallization process of copper foil, while studies on the effect of annealing atmosphere on abnormal grain growth behavior are relatively rare. It is necessary to carry out more studies on the effect of annealing atmosphere on grain growth behavior to understand the recrystallization mechanism of metal. Here, we found that introduction of ethanol in pure argon annealing atmosphere will cause the abnormal grain growth of copper foil. Moreover, the number of abnormally grown grains can be controlled by the concentration of ethanol in the annealing atmosphere. Using this technology, the number of abnormally grown grains on the copper foil can be controlled to single one. This abnormally grown grain will grow rapidly to decimeter-size by consuming the surrounding small grains. This work provides a new perspective for the understanding of the recrystallization of metals, and a new method for the preparation of large-area single-crystal copper foils.

## 1. Introduction

Single-crystal metal foils with various facet indices have attracted considerable interest due to their potential applications in electronics [1,2], catalysis [3,4], and crystal epitaxy [5,6,7,8,9]. The single-crystal metals are usually obtained by cutting the bulk crystal grown by the Czochralski or Bridgman methods [2,10], or epitaxial deposition on the surface of other single-crystal inorganic substrates with small lattice mismatch [11,12,13]. These methods only lead to few kinds of crystal facets with limited size, and the price is very expensive.

Recently, a technology has been developed to prepare large-area single-crystal copper foil by abnormal grain growth during the annealing of polycrystalline copper foil [14,15,16]. The abnormal grain growth is affected by multiple factors, such as driving force [17] and temperature [18,19,20]. The tension can activate the grain boundary energy of Cu foil and promote its abnormal grain growth to single crystals [17]. The difference of the main force for grain growth in polycrystalline metal will result in abnormal grown grain with different facet index. For example, when the surface energy as the main diving force, the abnormal grain growth trends to formation of Cu (111) with minimum surface energy [21]. When the thermal stress that arises as a result of interfacial contact as the major driving force for grain growth, abnormal grown grain with a high-index facet can be obtained [19]. High temperature (close to the metals’ melting temperatures) can accelerate the abnormal grain growth [21]. A static gradient of temperature can promote the continuous growth of the abnormal grains [18,19,20].

The annealing atmospheres also have an important effect on the recrystallized texture and abnormal grain growth of metal. Hydrogen, as a reductive gas, can reduce the formation energy of vacancies in the bulk which is highly critical for grain rotation [21], and favors the triggering of the abnormal grain growth at high temperature [22]. Therefore, hydrogen has often been used in the research works of preparing large-size single-crystal metals, based on abnormal grain growth [17,18,19,20,21,23]. Inert gas, usually be used as a diluting or shielding gas, has little promotion effect on the abnormal grain growth of metal; similar to the way in which annealing copper foils in pure argon gas can’t obtain large-size abnormal grown grains [20,21]. However, there are few studies on the influence of other annealing atmosphere on the recrystallization process of copper foil. It is necessary to study the effect of more annealing atmosphere on abnormal grain growth to understand the recrystallization mechanism of metals.

Ethanol is often used in experiments, such as in cleaning substrates or quartz tubes, and can also be used as carbon source to prepare graphene on copper foils at low temperatures [24]. Studying the influence of ethanol on the recrystallized process of copper foil not only provides a new perspective for understanding the recrystallization mechanism of copper foil, but also provides a new understanding of the experiment results with ethanol involved.

## 2. Materials and Methods

### 2.1. Annealing Copper Foils

We used the typical contact-free method of annealing copper foils. The annealing of copper foils was carried out in a split tube furnace (Anhui BEQ Equipment Technology Co., Ltd., Hefei, China). The commercially available copper foils (25 μm-thick and 46 μm-thick foils, 99.95% purity from Chinalco Shanghai Copper Co., Shanghai, China; 70 μm-thick foil, 99.98% purity from Lingbao Jinyuan Zhaohui Copper Co., Lingbao, China; 80 μm-thick foil, 99.9% purity from Nilaco Co., Tokyo, Japan) were vertically fixed by home-made quartz holders. Then, the quartz holder with copper foil was loaded in the center of the furnace. Different amounts of ethanol (Chengdu Kelong Chemical Co., Chendu, Ltd., China), drawn by a pipette, were loaded in a ceramic boat, which was then placed at the gas inlet side of quartz tube. After the reactor was evacuated to a pressure of <5 Pa, Ar (99.999% purity) was introduced to break the vacuum to normal pressure. Subsequently, the furnace was heated up to 1060 °C within 90 min in a 1000 sccm Ar flow and then maintained for 30 min. For 70 μm-thick and 80 μm-thick copper foils, the annealing temperature and the annealing time were increased to 1070 °C and 5 h, respectively. After annealing, the furnace was opened for fast cooling. When no ethanol was introduced into the reactor, the same number and size of copper foil was load in the furnace, and then the same annealing process was performed.

### 2.2. Sample Characterization

The crystal orientation of the prepared samples was investigated by electron backscatter diffraction (EBSD, Oxford symmetry Camera in combination with GeminiSEM 300, Carl Zeiss AG, Oberkochen, Germany) and X-ray diffraction (XRD, X Pert powder). The morphology and surface of the samples were investigated by atomic force microscope (AFM, Dimension Edge, Bruker, Billerica, MA, USA), a scanning electron microscope (SEM, JSM-7800F, JEOL, Tokyo, Japan), and an optical microscope (OM, Nikon DS-Fi2, Tokyo, Japan).

## 3. Results and Discussion

In this study, we found that ethanol can effectively induce the abnormal grain growth of copper foil during high temperature annealing. The number of the abnormally grown grains is very sensitive to the amount of ethanol in the annealing atmosphere. By controlling the amount of ethanol introduced in the annealing atmosphere, a decimeter-scale grain can be obtained on the copper foil. The EBSD and XRD were used to confirm the crystal orientation of as-obtained large grains. The as-prepared single-crystal grains are also applicable to the epitaxial growth of high-quality graphene.

**Figure 1:**
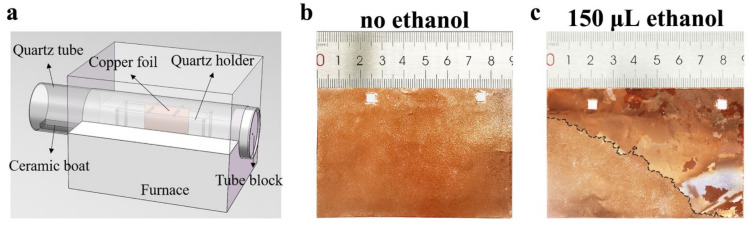
(**a**) Schematic diagram of contact-free annealing configuration, from which the copper foil is suspended in the middle of furnace. (**b**,**c**) are the photographs of Cu foil annealed in argon atmosphere without and with 150 μL ethanol, respectively. The black dashed line in (**c**) corresponds to the grain boundaries between large grains and polygranular regions.

To highlight the influence of ethanol, we used the typical contact-free method of annealing copper foils, to eliminate or minimize the influence of other factors, such as strain energy and interface energy that arise from the interfacial contact [19]. The contact-free annealing schematic is depicted in Figure 1a. The Cu foil, fixed on the quartz holder, is suspended in the middle of furnace, as reported in other work [25]. Similar to the previous CVD graphene growth route, using liquid carbon source [26], the ethanol loaded in a ceramic boat was placed at the gas inlet side of quartz tube. A porous ceramic tube block was placed at the gas outlet side of the tube for blocking heat radiation generated from the tube’s center. Before the furnace heated up, the reaction chamber was first evacuated to low pressure, and then backfilled with argon gas to normal pressure. After these operations, most of the ethanol was removed from the reaction chamber. The ethanol involved in the recrystallization process of copper foils is mainly the ethanol adsorbed on the inner surface of the chamber, the outer wall of the quartz boat, the porous ceramic tube block, the ceramic boat, and the surface of the copper foil. The amount of ethanol adsorbed in the reaction chamber is related to the amount of ethanol introduced into the chamber. Therefore, we indirectly control the amount of ethanol absorbed by introducing the amount of ethanol.

To better illustrate the effect of ethanol on abnormal grain growth, we first annealed the copper foils in pure argon gas. The results showed no obvious abnormal growth of millimeter-level and above grains were found (as shown in Figure 1b), which was consistent with previously reported results [21]. We define this state as the initial state. In the study of the influence of ethanol on the recrystallization process of copper foil, we first introduced 150 μL ethanol into the reaction chamber in the manner shown in Figure 1a. As reported by previous research, the grain boundaries can be observed with the naked eye when the size of individual grains reaches the centimeter scale [21,27,28]. The centimeter-level grains can be clearly seen with the naked eye on copper foil annealed in the ethanol atmosphere. This result shows that ethanol can affect the recrystallization process of copper foil at elevated temperatures.

As mentioned above, most of the ethanol introduced was removed from the reaction chamber during the evacuation stage. The ethanol involved in the recrystallization process of copper foils was the ethanol desorbed from the ethanol adsorbed in the reaction chamber. The amount of ethanol involved in the reaction was very little. That is to say, the abnormal grain growth of copper foil is very sensitive to ethanol at a high annealing temperature.

**Figure 2:**
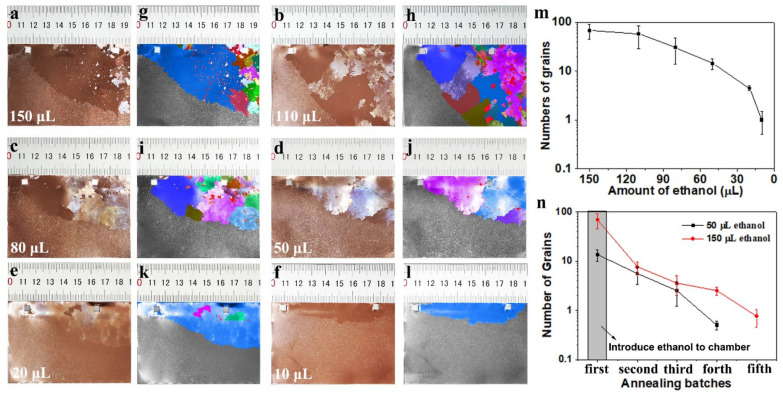
(**a**–**f**) and (**g**–**l**) are the photographs of copper foils annealed under different amounts of introduced ethanol, and their corresponding false-color images, respectively. (**m**) Statistics chart of the number of millimeter-sized grains and above on the copper foils, annealed under different amounts of ethanol introduced. (**n**) Statistics chart of number of grains on copper foils obtained in different annealing batches after different amounts of ethanol were introduced.

To further illustrate the influence of ethanol on the grain growth behavior of Cu foil, we studied the variation of the number of abnormal growing grains with the amount of ethanol introduced. Although most of the ethanol was removed during the process of evacuating the tube reactor to a low pressure, as the total amount of ethanol introduced increased, the concentration of ethanol remaining in the reactor also increased, relatively. Figure 2a–f are the photographs of copper foils annealed under different amount of introduced ethanol. In order to distinguish the millimeter-size and above grains, the photographs were treated with false-color imaging, assisted by OM (as shown in Figure 2g–l). The OM was used in refractive mode, and via the difference in reflection, the Cu grains and the grains boundaries could be identified. It can be clearly seen from the photographs and false-color images that, when 150 μL of ethanol was introduced, there were more than 20 grains with sizes of the centimeter scale, and the others are millimeter-sized crystal grains. Due to the differences in the critical barriers for abnormal grain growth at different positions of the copper foil, the abnormally grown grains appear preferentially at the positions with lower barriers, while appearing relatively late at the positions with higher barriers [21]. Driven by the kinetics, the early appearing grains grew faster by consuming neighboring small grains [14]. Therefore, after 30 min of annealing, those abnormally grown grains which appeared later were surround by the fast-growing grains (as shown in Figure 2a).

From the photographs (Figure 2a–f) and the statistical graph of the change in the number of grains with the amount of ethanol introduced (as shown in Figure 2m), we observed that the total number of abnormally grown grains decreases rapidly as the amount of ethanol introduced gradually decreases. When the amount of introduced ethanol was reduced to 10 μL, only one large grain can be seen on the obtained copper foil (see Figure 2f or Figure 2l). When continuing to reduce the amount of introduced ethanol, no abnormal growth of crystal grains was found on the annealed copper foil, because the amount of ethanol involved in the reaction was lower than the critical value for inducing abnormal grain growth.

Note that, each time the ethanol was introduced to the tube reactor, multiple batches of annealing experiments under the same conditions were required to eliminate the effect of the residual ethanol in the reactor on the recrystallization of copper foil. When there was no abnormal grain growth on the annealed copper foil for several consecutive batches, the tube reactor was considered to be restored to its initial state. When the introduced ethanol is 10 μL (first batch), no abnormally grown grain was observed from the second batch. The number of annealing batches required to eliminate the residual ethanol in the reactor increases as the amount of introduced ethanol increases. Figure S1 shows the photographs of copper foils obtained from different annealing batches and the corresponding false-color images. It can be found that, as the annealing batch increases, the number of abnormally grown grains gradually decreases. A single large grain can be obtained in the fourth batch when the introduced ethanol is 50 μL. We compared the variation of the number of abnormally grown grains with annealing batches when ethanol was introduced at 50 μL and 150 μL, respectively (as shown in Figure 2n). The result showed that the average number of grains obtained in the first batch, at 150 μL ethanol, was much higher than that obtained at 50 μL ethanol; however, in the second batch, the average number of grains obtained in the two cases dropped to the same order of magnitude. This indicates that the introduced ethanol was almost consumed in the first batch, resulting in the residual ethanol in the reactor at the same magnitude in the second batch.

We observed that the single abnormally grown grain, which can be regarded as the first abnormally grown grain, preferentially appeared at the upper edge region of foils when the concentration of ethanol was very low (as shown in Figure S2). This may be because grains near the cut edge have higher stored energy, which can accelerate the abnormal grain growth [21,29]. Moreover, the temperature and the concentration of ethanol near the upper region of furnace were relatively higher at atmosphere pressure due to the thermal buoyancy [30]. As the amount of ethanol increased, the concentration of ethanol on the copper foil surface also increased correspondingly. At this time, abnormally grown grains were also observed in the middle and non-edge regions of copper foil, which further indicated that ethanol plays a key role in the recrystallization process of copper foils.

During the 30 min annealing process, the abnormally grown grains show two different growth types, one is that the large grains are adjacent to each other, and another is that some grains are wrapped in a large grain (as shown in Figure 2). To better understand the grains growth behavior, we studied the crystal orientation of the representative grains by EBSD, which is a SEM-based method for analysis of crystal structure of crystalline materials. The results showed that the abnormally grown grains obtained by two growth types have the vicinal surface (Figure S3). That is to say, after 30 min of annealing, the abnormally grown crystal grains gradually formed multiple large crystal grains by consuming the surrounding small grains. It took a long time to consume these large grains to form a single grain [21]. During the annealing process, if the number of the abnormally grown grains can be reduced to a single grain, the polycrystalline copper can be transformed into a large-sized, single-crystal grain.

**Figure 3:**
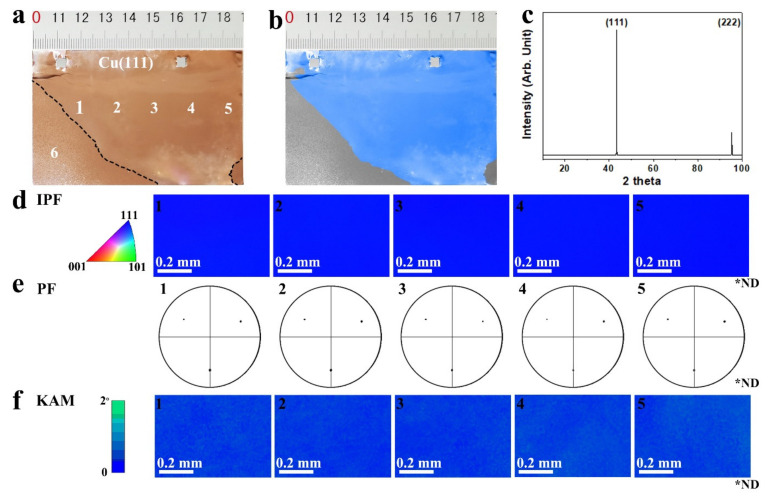
(**a**,**b**) are the photographs of the annealed copper foil with a decimeter-sized abnormally grown grain, respectively. (**c**) The XRD 2θ scan spectrum of the large grain. EBSD IPF maps in the normal direction (**d**); (001) pole figures (**e**); and KAM maps (**f**) of the single large grain of copper foil collected at the corresponding positions marked in (**a**). (ND, normal direction).

By carefully controlling the amount of ethanol introduced into the tube reactor at 10 μL and appropriately extending the annealing time of the copper foil to 2–4 h, we obtained a single abnormally grown grain with decimeter level (see Figure 3a,b, where an irregular grain of about 9 × 6 cm^2^ can be seen). The EBSD and XRD were used to analyze the crystal orientation of large grains. The XRD 2*θ* scan spectra only showed two characteristic peaks of (111) and (222) crystal plans (Figure 3c). Other characteristic peaks of other crystal plans were not observed, indicating that the polycrystalline copper foil was transformed into a large grain with a (111) texture. To further illustrate the distribution of the crystal texture and test the crystallographic orientations of this large grain, both in the normal direction and the in-plane direction, we conducted EBSD measurements at five different regions, marked in Figure 3a. The inverse pole figure (IPF) maps, in normal direction, showed a uniform blue color (Figure 3d), verifying the (111) facet index. The five regions had the same in-plane crystallographic orientation as those in the (001) pole figures (Figure 3e). All the kernel average misorientation (KAM) maps showed a small misorientation (less than 1°) between the measured points and their neighbors (Figure 3f), confirming that the abnormally grown large grain was a homogeneous single crystal with index (111) facet.

**Figure 4:**
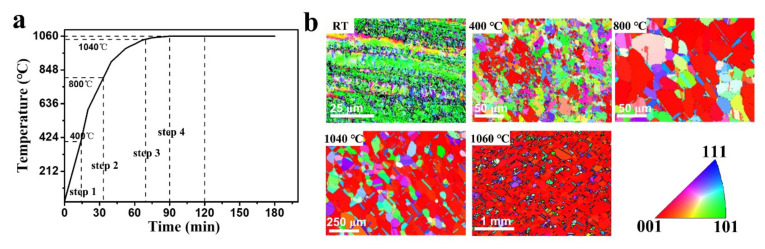
(**a**) Annealing sequence of copper foils. (**b**) EBSD IPF maps in the normal direction of copper foils at the different annealing temperatures shown in (**a**).

To further illustrate the texture evolution and the grain growth behavior of copper foil, we annealed the copper foil using the steps shown in Figure 4a. It can be found from the IPF maps (shown in Figure 4b) that the grains, which have an initial texture of (110) and some additional other textures, gradually recrystallized to (100) texture as the annealing temperature increased. When the temperature reached 1060 °C, most of the grains were (001) facet and vicinal facet, before the abnormal grain growth, which agreed well with the EBSD result (as shown in Figure S4)—conducted at the polycrystalline regions marked in Figure 3a. This is because the stored strain energy in some cold rolled polycrystalline Cu foils drives most grains to rotate to (001) crystal orientation with a high density of low-angle grain boundaries around (001) grains [15,19,31]. As the annealing time increased, some grains began to abnormally grow to decimeter-sized grains with (111) crystal orientation.

**Figure 5:**
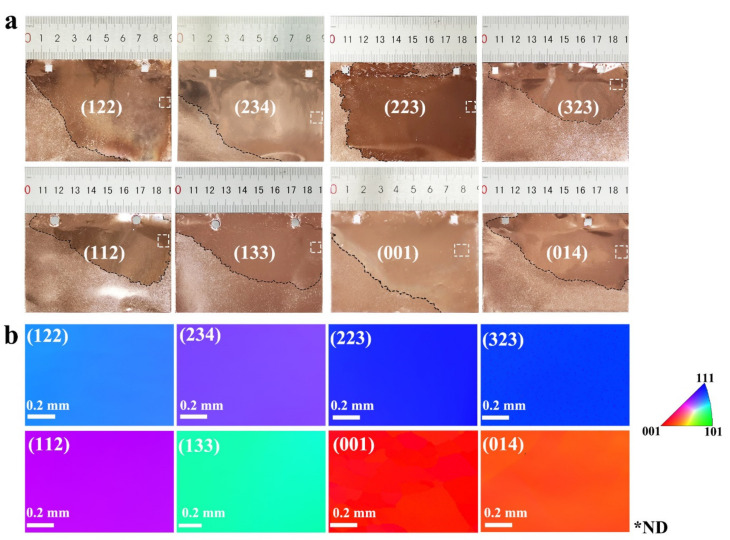
(**a**) Photographs of eight representative annealed copper foils with a decimeter-sized, abnormally grown grain. (**b**) The corresponding EBSD IPF maps in the normal direction of these large grains, collected at the corresponding positions marked with white dotted box in (**a**). Black dash line in (**a**) corresponds to grain boundaries between large grain and polygranular regions. ND, normal direction).

To verify the feasibility of this method, we repeated the annealing procedure that can prepare a single centimeter-level, abnormally grown grain on multiple pieces of copper foils. About 11 kinds of abnormally grown grains with different crystal orientations, at decimeter-size, were obtained. Figure 5a shows eight representative kinds of copper foil with a typical abnormally grown, decimeter-sized grains and different facet indices. The black dash line in Figure 5a corresponds to the grain boundary between large grain and polycrystal regions. The distinct color in the corresponding EBSD IPF maps (Figure 5b) further confirm that the abnormally grown large grain on annealed copper foils have the characteristics of different facet indices. The XRD 2*θ* scan spectra (Figure S5a) show the characteristic peaks of (111) facet and (100) facet, while the 2*θ* peak for other facets are not displayed because they are out of the XRD scan range [20]. The statistics of EBSD measurement results of the 41 pieces of the large-area single-crystal grain showed that the (111) facet was the most frequently obtained crystal plane in general (Figure S5b). As the (111) vicinal surface, (223), (122), and (323) facets were often obtained with probabilities of 20%, 17%, and 12%, respectively. The probability of obtaining other facets, such as (100), (014), and (236), were less than 6%.

The pre-oxidized copper foils were also used to anneal in pure argon and ethanol atmospheres. The copper foils were first oxidized at 200 °C in air for 5 min, 10 min, and 30 min, respectively, then annealed under the same conditions as the non-oxidized copper foils. The results show that no abnormally grown grains were observed in pre-oxidized copper foils annealed in pure argon gas, while the pre-oxidation copper foils annealed in ethanol atmosphere show the same growth behavior of crystal grains as non-oxidized copper foils. Therefore, the influence of surface oxidation of copper foil on the growth behavior of grains can be eliminated. The ethanol is the main factor for the texture evolution and the grain growth behavior of copper foil.

In order to study the influence of ethanol concentration on the recrystallization process of copper foil with its thickness, we annealed other three different copper foils (46 μm, 70 μm, and 80 μm) (Figure S6). It was found that the influence of ethanol on the process of abnormal grain growth decreased with the increase of the copper foil thickness, when the amount of introduced ethanol was the same. Such as the abnormally grown grains on the 46 μm foil and 25 μm foil had a similar growth behavior, but the number of abnormally grown grains on the 46 μm foil was less than that on 25 μm foil under the same conditions. For copper foils with a thickness of 70 μm and 80 μm, there is no abnormal grain growth after annealing at 1060 °C for 5 h, even if the amount of ethanol introduced is 150 μL. Centimeter-sized, abnormally grown grain was observed on the 70 μm foil after annealing at 1070 °C for 5 h (the amount of ethanol kept 150 μL) (Figures S6 and S7), while no abnormally grown grains were observed on 80 μm foil. Moreover, to verify the potential applications of the as-obtained, large-sized, single-crystal grains, we used these annealed copper foils with decimeter-sized, single-crystal grain as the catalytic substrate to prepare graphene (see the graphene growth in Supplementary Materials and Figure S8). From the SEM images of graphene domains, it can be seen that the oriented graphene domains grew epitaxially on (112), (223), and (323) crystal planes other than the (111) crystal plane (as shown in Figure S9).

## 4. Conclusions

In conclusion, we have demonstrated that ethanol can affect the recrystallization process of copper foil. The abnormal grain growth appeared on the polycrystalline copper foil when ethanol was introduced into the pure argon annealing atmosphere. The number of abnormally grown grains on the copper foil can be controlled by the amount of ethanol introduced into the reactor. Moreover, the grain growth behavior is very sensitive to the amount of ethanol in the annealing atmosphere. Multiple annealing batches are required to eliminate the effect of residual ethanol in the reactor on the grain growth behavior, when the ethanol was introduced into the reactor. Furthermore, a large-sized, abnormally grown grain can be obtained on the polycrystalline copper soil by reducing the amount of ethanol introduced into the reactor to 10 μL and extending the annealing time to 2–4 h. We found that the influence of ethanol on the grain growth behavior of Cu foils decreases with the increase of the copper foil thickness, at the same concentration level of ethanol. The obtained large-area, single-crystal grain can be used as catalyst to epitaxially grow high-quality graphene.

## Supplementary Materials

The following are available online at https://www.mdpi.com/article/10.3390/nano11113069/s1, Figure S1: the photographs of copper foils obtained from different annealing batches and the corresponding false-color images, respectively, Figure S2: the photographs of annealed copper foil with only one abnormally grown grain and the corresponding false-color images, respectively, Figure S3: the photographs of two copper foils with different type of abnormally grown grains and the EBSD IPF maps in the normal direction of the single large grain, Figure S4: EBSD IPF maps in the normal direction (a) and (001) pole figures of the annealed copper foil collected at the 6th region marked in Figure 3a, Figure S5: XRD 2θ scan spectra of the five kinds of abnormally grown single-crystal grain and Pie chart of proportions of facets of 41 pieces of annealed copper foils with a decimeter-size single crystal grain, Figure S6: the photograph of annealed copper foils with thickness, Figure S7: The photograph of annealed 70 μm thick copper foil and EBSD measurement results, Figure S8: AFM images of large-area abnormally grown grains surfaces of Cu foil, Figure S9: the SEM images of epitaxial growth of graphene on abnormally grown large grain of Cu foils.
