# Supplementary material for: The Effect of Ethanol on Abnormal Grain Growth in Copper Foils
Source: Nanomaterials (Basel). 2021 Nov 15;11(11):3069. doi: 10.3390/nano11113069 (PMC8625376; doi:10.3390/nano11113069)
Supplement: Supplementary file 1 [file nanomaterials-11-03069-s001.zip › nanomaterials-1434867-supplementary.pdf]

nanomaterials

Supplementary Materials

The Effect of Ethanol on Abnormal Grain Growth in Copper Foils

Zhancheng Li [1], Yongna Zhang [1], Yinwu Duan [2], Deping Huang [1,*] and Haofei Shi [1,*]

[1] Chongqing Institute of Green and Intelligent Technology, Chinese Academy of Sciences, Chongqing 400714, China; lizc@cigit.ac.cn (Z.L.); zhangyn@cigit.ac.cn (Y.Z.)
[2] Chongqing Engineering Research Center of Graphene Film Manufacturing, Chongqing 401329, China; duanyw@moxigroup.com
* Correspondence: huangdeping@cigit.ac.cn (D.H.); shi@cigit.ac.cn (H.S.)

The Effect of Concentration of Ethanol on the Grain Growth Behavior

The grain growth behavior of copper foil is very sensitive to the concentration of ethanol in the atmosphere. Ethanol is introduced into the tube reactor at the sample loading stage. Then the tube reactor was evacuated to a pressure of < 5 Pa. That is to say that the concentration of ethanol in annealing atmosphere is very low, about ppm level. Each time the ethanol was introduced to the tube reactor, multiple batches of annealing experiments in pure Ar gas flow are required to eliminate the effect of the residual ethanol on the grain growth behavior. Figure S1 show the photographs of copper foils obtained from different annealing batches and the corresponding false-color images after introducing 50 μL to the tube reactor. We observed that that as the annealing batch increases, the number of abnormally grown grains gradually decreases. And only a single large grain can be obtained on copper foil in the last batch.

Using this technology, we have controlled the number of abnormally grown grains on the copper foil to single one (Figure S2). We observed that these grains almost appear at the upper edge regions. This is because that the grains near the cut edge have higher stored energy than other grains [1,2], which can accelerate the abnormal grain growth.

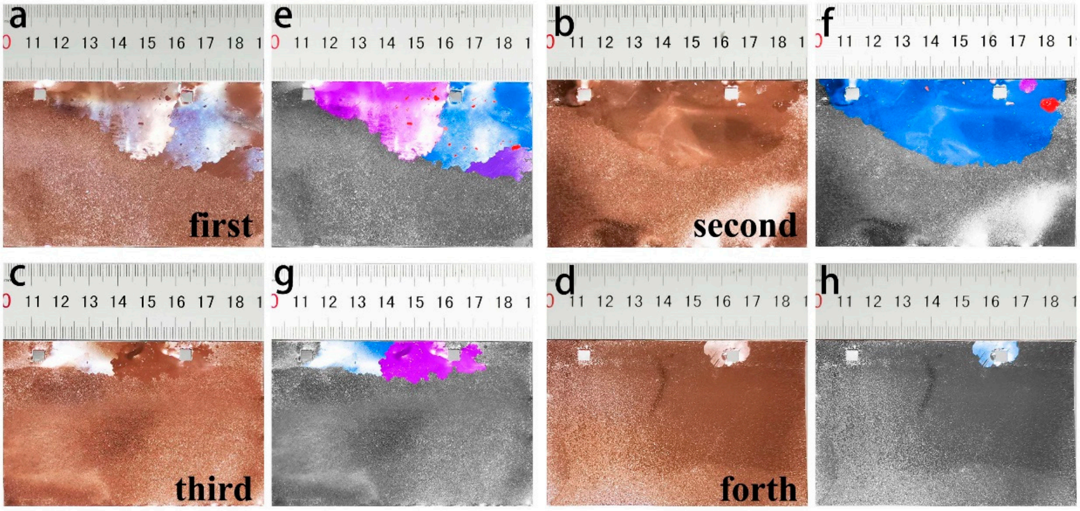

Figure S1. (a−d) and (e−h) are the photographs of copper foils obtained from different annealing batches and the corresponding false-color images, respectively. The amount of ethanol introduced in the first batch is 50 μL.

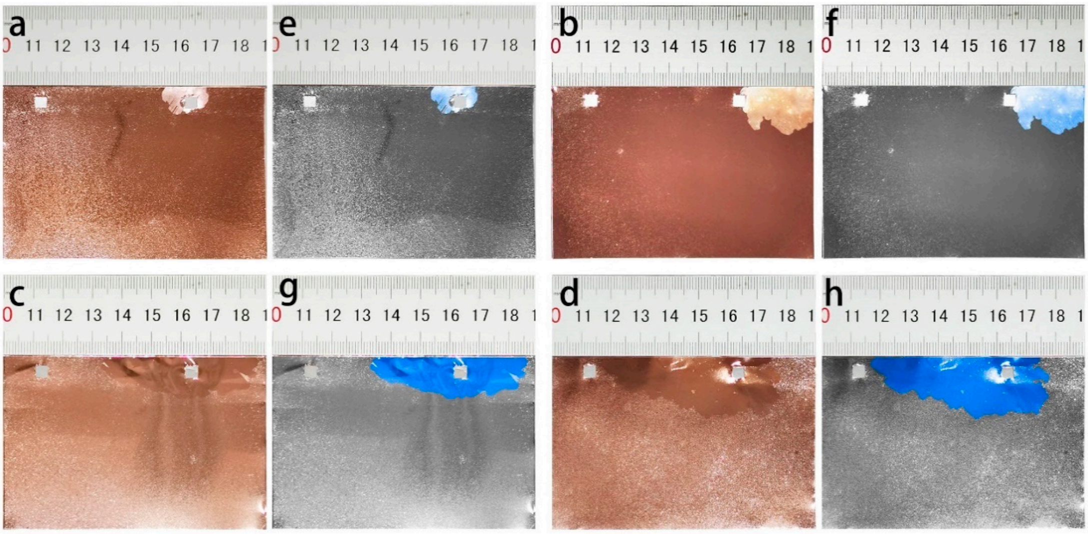

Figure S2. (a–d) and (e–h) are the photographs of annealed copper foil with only one abnormally grown grain and the corresponding false-color images, respectively.

Different Growth Types of Abnormally Grown Grains

After 30 min annealing in ethanol atmosphere, some small grains were wrapped in a large gain, while other large gains are adjacent to each other. To better illustrate the grain growth behavior, the EBSD was used to analyze the crystal orientation. From the photographs and the EMSD IPF maps, we can clearly see that whether they are adjacent to each other or wrapped in one another, these grains have the vicinal surface, such as (111) facet and (122) facet, (133) facet and (122) facet. The grains with vicinal surface have low misorientation angles of grain boundaries. The mobility and energy of high angle grain boundaries is much higher than that low angle grain boundaries. So, the abnormally grown grains cannot consume each other in a short time annealing.

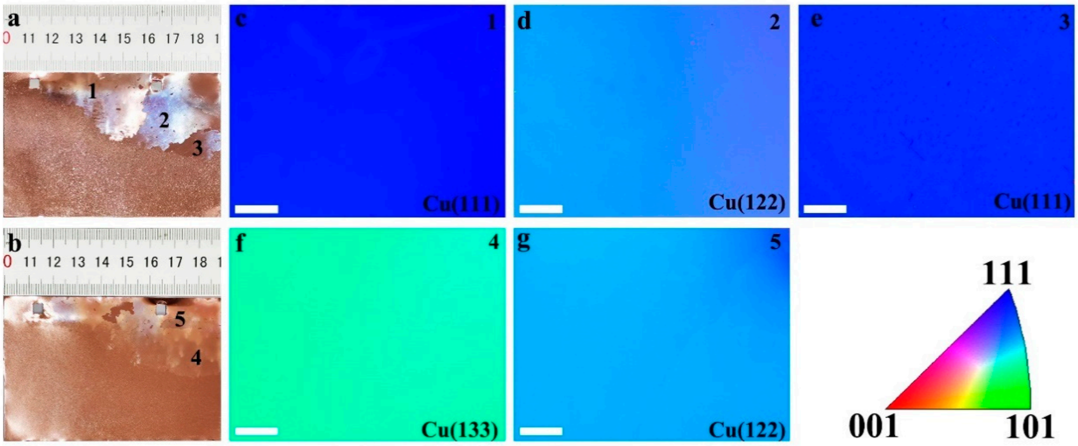

Figure S3. (a) and (b) are the photographs of two copper foils with different type of abnormally grown grains, the large grains are adjacent to each other (a) and some grains are wrapped in another grain (b). (c–g) EBSD IPF maps in the normal direction of the single large grain of copper foils collected at the corresponding positions marked in (a) and (b). The scale bar is 200 μm.

The Texture Evolution of Cu Foil during Ethanol Annealing Atmosphere

To examine the texture evolution and grain growth behavior of Cu foil during ethanol annealing atmosphere, we annealed the copper foils using the steps shown in Figure 4a. It can be observed that the grains gradually transformed from the initial main

texture of (110) to main texture of (100) as the annealing temperature increases (Figure 4b). When the temperature reaches 1060 °C and before the abnormal grain growth, most grains are the (001) and its vicinal surface, and few other crystal orientations, which agree well with the EBSD results (see Figure S4) conducted at the polycrystalline region marked in Figure 3a. The distribution of misorientation angles between grains shows that most grains have low angle grain boundaries and a small part of grains have higher angle grain boundaries. As the annealing time increases, the grains with higher grain boundaries grow faster by consuming surrounding small gains [1], thereby resulting in the abnormal grain growth.

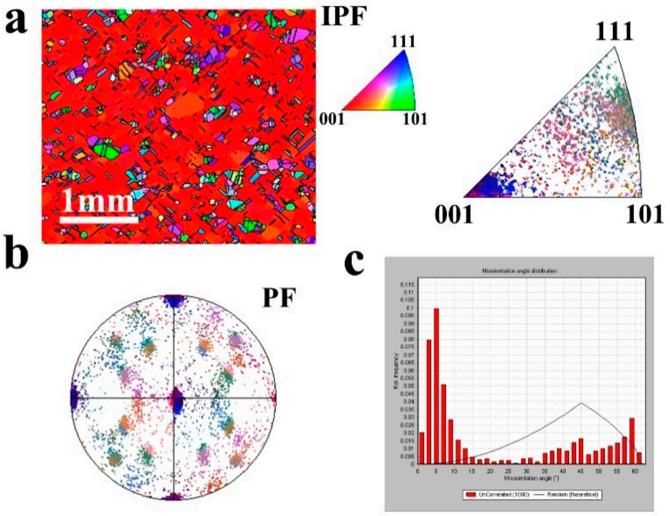

Figure S4. (a) and (b) are the EBSD IPF maps in the normal direction (a) and (001) pole figures of the annealed copper foil collected at the 6th region marked in Figure 3a. (c) Distribution of misorientation angles of grain boundaries.

XRD Data and the Statistics of Crystal Orientations

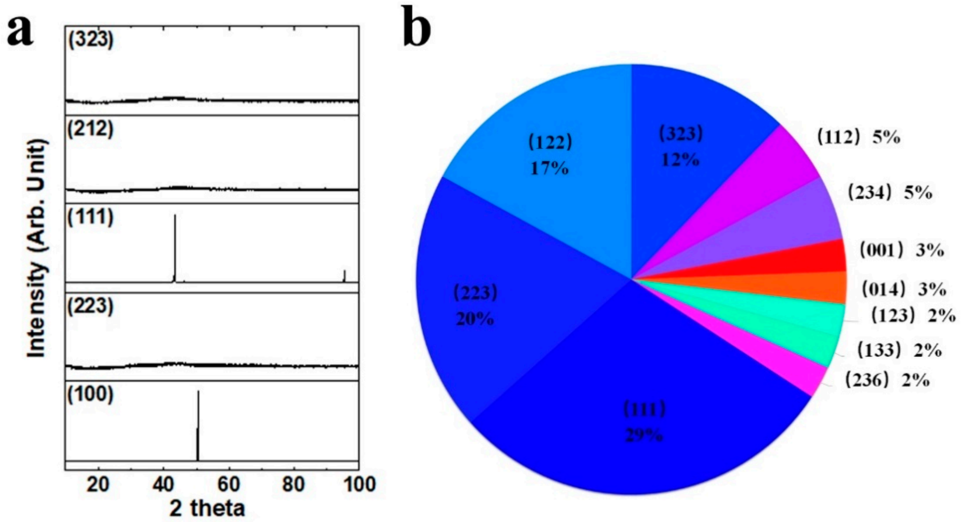

Figure S5. (a) XRD 2θ scan spectra of the five kinds of abnormally grown single-crystal grain. (b) Pie chart of proportions of facets of 41 pieces of annealed copper foils with a decimeter-size single crystal grain.

The Effect of Ethanol on Grain Growth of Different Thickness Cu Foils

We have annealed another three different thickness copper foils (46 μm-thick, 99.95% purity from Chinalco Shanghai Copper Co., 70 μm-thick, 99.98% purity Lingbao Jinyuan Zhaohui Copper Co., 80 μm-thick, 99.9% purity from Nilaco Co.). We found that the number of abnormally grown grains decreases with the increase of Cu foil thickness at the same concentration of ethanol. The ethanol can relatively easily to induce the abnormal grain growth on 46 μm-thick copper foils (see Figure S6). For 70 μm-thick and 80 μm-thick copper foils, it will need more amount of introduced ethanol, higher annealing temperature and longer annealing time (Figure S6). A centimeter-size gran can be found on the 70 μm-thick copper foil after annealing at 1070 °C for 5 h with the amount of introduced ethanol of 150 μL (as shown Figure S6 and S7), While no abnormally grown grains on the 80 μm-thick copper foil under the same annealing process (see Figure S6).

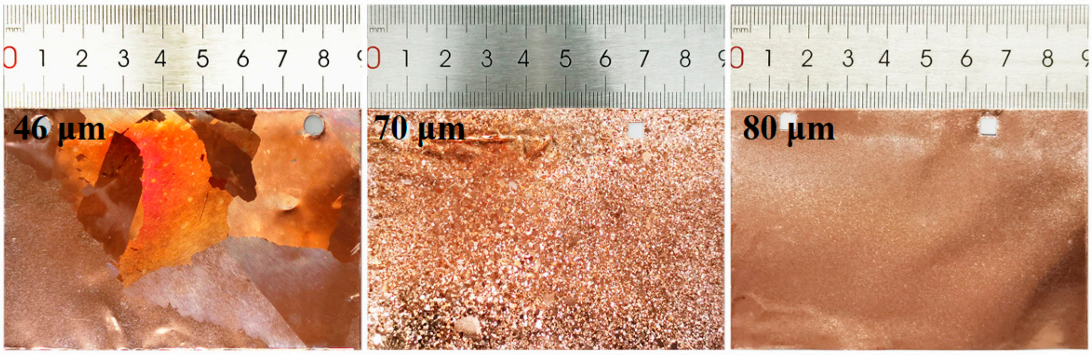

Figure S6. The photograph of annealed copper foils with thickness. The 46 μm thick copper foil was annealed at 1060 °C for 30 min in the amount of introduced ethanol 80 μL. The 70 μm and 80 μm thick copper foils were annealed at 1070 °C for 5h in the amount of introduced ethanol 150 μL.

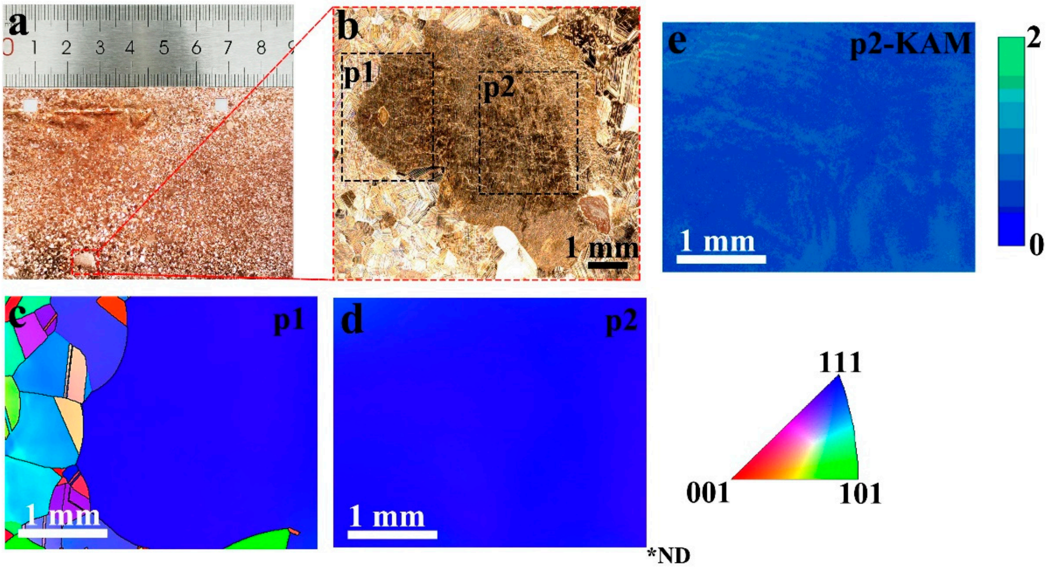

Figure S7. (a) The photograph of annealed 70 μm thick copper foil. (b) The optical microscope image of the abnormal grain growth region marked in (a). (c)–(d) are the EBSD IPF maps in the normal direction two different regions marked in (b). (e) KAM maps of the p2 region marked in (b).

AFM images of the large-area grain before and after electropolished

We used the as-obtained large-area single crystal grains with (112), (111), (323), and (223) facets as substrate to grow graphene. Before the graphene growth, the substrates were first electropolished in a H_3PO_4 solution by applying a constant voltage (about 0.8 V) for 16 min. The single crystal substrate was used as a working electrode, and a

polycrystalline copper foil as a counter electrode. The AFM measurement results show the large-size single-crystal grain have many rolling lines in the surface with an RMS roughness of 94.35 ± 2.95%. After electropolishing, most of these lines on the surface were removed. The final single-crystal grain had an ultra-flat surface with an RMS roughness of 1.84 ± 0.53%. (as shown in Figure S8) Then the electropolished substrate was annealed at 1045 °C for 20 min in a 20 sccm H_2 gas flow and 500 sccm Ar gas flow, and at atmospheric pressure. Subsequently, a dilute CH_4 gas (1% diluted in H_2) at a flow rate of 0.75 sccm was introduced to the quartz tube for 30 min. After growth, the furnace was opened for fast cooling.

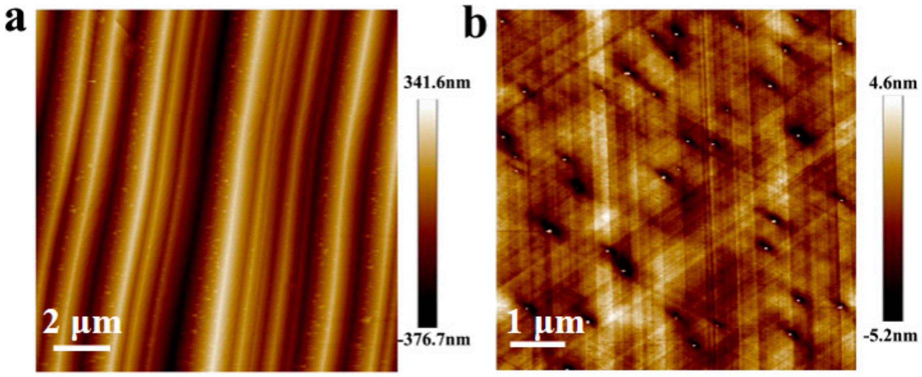

Figure S8. AFM images of large-area abnormally grown grains surfaces of Cu foil: (a) annealed, (b) electropolished.

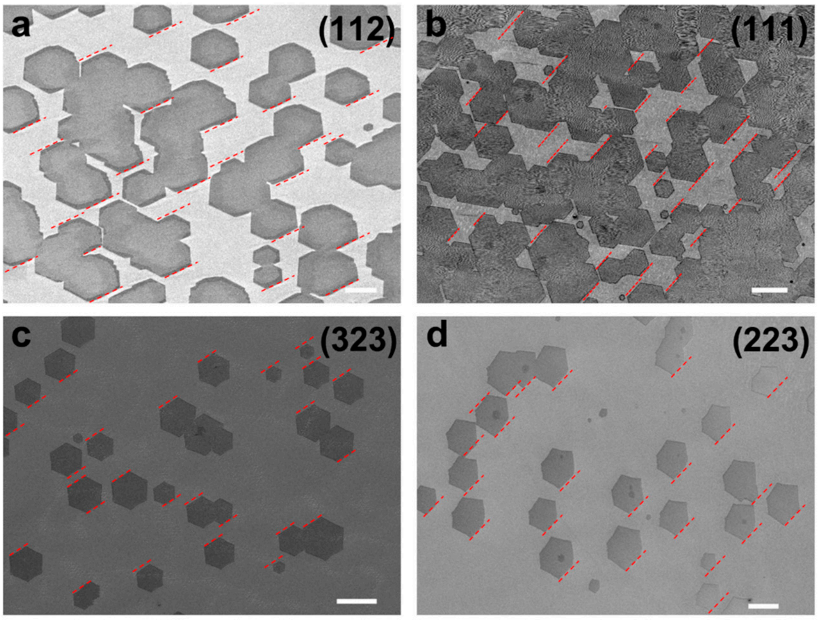

Figure S9. Epitaxial growth of graphene on abnormally grown large grain of Cu foils. (a–d) are the SEM images of unidirectionally aligned graphene domains on (112), (111), (323), and (223) facets, respectively. The scale bar is 10 μm.

References

1. Jin, S., Huang, M., Kwon, Y., Zhang, L.N., Li, B.W., Oh, S., Dong, J.C., Luo, D., Biswal, M., Cunning, B.V., Bakharev, P.V., Moon, I., Yoo, W.J., Camacho-Mojica, D.C., Kim, Y.J., Lee, S.H., Wang, B., Seong, W.K., Saxena, M., Ding, F., Shin, H.J. & Ruoff, R.S. Colossal grain growth yields single-crystal metal foils by contact-free annealing. *Science* **2018**, *362*, 1021–1025.
2. Wright, S.I., Nowell, M.M. & Field, D.P. A Review of Strain Analysis Using Electron Backscatter Diffraction. *Microsc. Microanal.* **2011**, *17*, 316–329.
